# Diabetes Mellitus Cured by Tincture of Cantharides

**Published:** 1835

**Authors:** 


### II.—ANALYTICAL.

I. *Diabetes Mellitus cured by Tincture of Cantharides.*—Professor Hall, of Baltimore, has published in the N. A. Archives for July last, the notes of a case of this obstinate disease, successfully treated with the tincture of cantharides. The patient was a youth of 17 years of age. The symptoms were not indicative of an inflammatory diathesis. Sulph. quin. phosph. ferri, etc. with animal diet were used without effect. He began by taking 20 drops of the tincture three times a day, gradually increasing the dose to 400, with but slight strangury! In about a month he was quite well.

